# *Candida glabrata*: A powerhouse of resistance

**DOI:** 10.1371/journal.ppat.1011651

**Published:** 2023-10-05

**Authors:** Seána Duggan, Jane Usher

**Affiliations:** Medical Research Council Centre for Medical Mycology at The University of Exeter, Exeter, United Kingdom; University of Maryland, Baltimore, UNITED STATES

## Introduction

*Candida glabrata (Nakoseomyces glabratus*) is a haploid, budding yeast that causes opportunistic nosocomial infections and is garnering increasing attention in line with its changing epidemiological importance. It is a commensal of the human mucosa, particularly oral, gastrointestinal, and vaginal epithelia, which predisposes to infection. The physical disruption of the epithelial layer is a major risk factor for systemic *C*. *glabrata* infection, which generally occurs in immunocompromised individuals. *C*. *glabrata* appears to lack the brute force traits of other *Candida* species such as filament formation leading to tissue damage and immune cell lysis but nevertheless leads the non-*Candida albicans* species in the number of patients it infects. Infections caused by *C*. *glabrata* are of great concern due to their propensity for drug resistance and limited treatment options. This article discusses the intrinsic and acquired factors contributing to *C*. *glabrata*’s increasing resistance to pharmaceutical intervention.

### 1: The evolutionary position of *Candida glabrata*

*C*. *glabrata* shares a recent common ancestor with several *Saccharomyces* species and belongs to a clade different from that of other Candida species (namely those that recode the CUG codon to serine). As a result of this evolutionary relatedness, most *Saccharomyces cerevisiae* genes have orthologues in *C*. *glabrata*, and the chromosomal structure in terms of gene order is largely conserved between the two. However, despite this general resemblance, there are several differences in terms of gene content, with *C*. *glabrata* having undergone increased gene loss [1]. Such reductive evolution could be connected to its adaptation as a commensal and opportunistic pathogen. Other significant differences include *C*. *glabrata*–specific gene expansions affecting cell wall organisation, where there are 6 copies of extracellular glycosylphosphatidylinositol-linked aspartyl proteases, 8 copies of alpha-1,3-mannosyltransferase involved in cell wall biogenesis, and a variable number of EPA (epithelial adhesin) genes located in subtelomeric regions [2].

**Fig 1:**
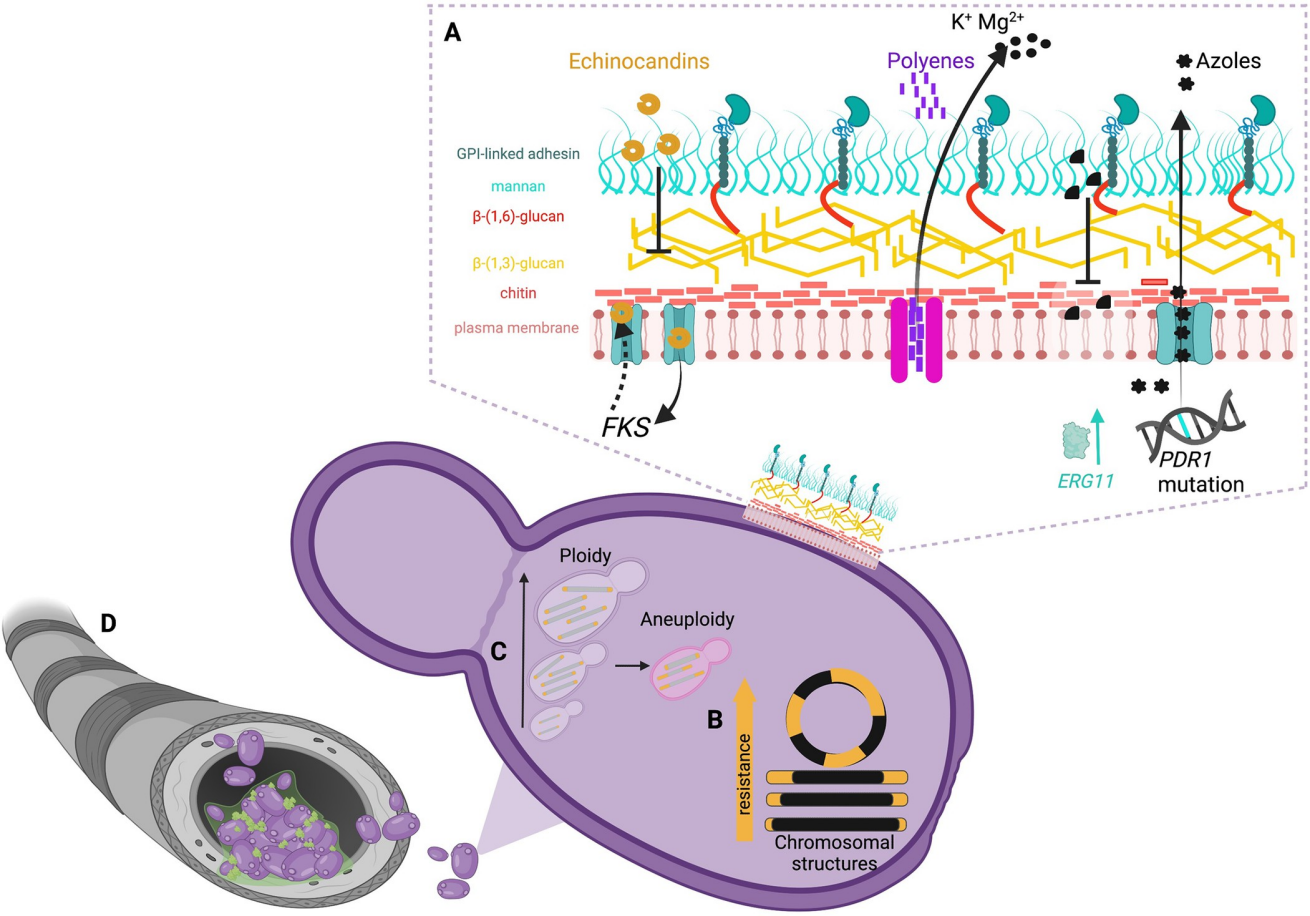
The arsenal employed by *C*. *glabrata* to resist pharmaceutical interventions. (**A**) The cell wall is the front line of battle between *C*. *glabrata* and antifungal drugs. Azoles target the plasma membrane, but resistance to azole drugs occurs through gain of function mutation in pdr1, which increases expression of drug efflux pumps. Polyenes bore into the plasma membrane paving the way for cytoplasmic contents, e.g., ions to leech into the extracellular space. Echinocandins target B-(1,3)-glucans. Resistance is consistently related to mutations in FKS genes, which normally govern B glucan synthesis. (**B**) Genomic flexibility drives adaptability and resistance in *C*. *glabrata*. Antifungal drug resistance correlates with increased telomere length and circularity of chromosomes. (**C**) Ploidy and aneuploidy drive genome plasticity by providing the framework through which the fungus makes genetic changes. (**D**) Biofilms adhere to biotic and abiotic surfaces, e.g., during mechanical ventilation. Due to varying cellular states and extracellular matrix, these entities are recalcitrant to therapeutics. Cells can slough off and result in systemic infection. This figure was created with Biorender.com.

The comparison of genomes under an evolutionary perspective is a powerful means by which to understand function, origin, and evolution of biological processes such as pathogenesis. Among its phylogenetic relatives, *C*. *glabrata* is notable by heightened genomic plasticity and genetic rewiring, which refers to redirection, redistribution, or duplication of genetic material [3] resulting in an altered genetic landscape (see Table 1 for definitions). Examples include chromosomal rearrangements, telomere dynamics, DNA damage response, and DNA mismatch repair systems [4]. Specifically, aneuploidy, gene gain and loss, chromosomal fusions, circulisations, and nonreciprocal translocations occur in *C*. *albicans* upon exposure to antifungal and anticancer drugs (Fig 1) [5]. These rearrangements permit rapid adaptation to and acquisition of drug resistance. Despite the potential perils associated with genomic instability, *C*. *glabrata* benefits through normal cell persistence and proliferation [6].

**Table 1:** Definitions of terms.

Terminology	
**aneuploidy**	addition or absence of one or more chromosomes
**circulisation**	ring structure, occurs when a chromosome breaks and ends fuse together
**chromosomal fusion**	hybrid chromosome formed by the joining of independent chromosomes
**segmental aneuploidy**	gains or losses of chromosomal segments
**telomere**	repetitive nucleotide sequences bookending linear chromosomes
**centromere**	point at which paired chromatids attach
**dicentric chromosome**	chromosome with 2 centromeres
**isochromosome**	unbalanced structural abnormality where chromosome arms mirror each other
**DNA mismatch repair**	corrects mistakes generated during DNA replication
**mating locus activity**	expression of or functional MAT a or MATα
**nonsynonymous mutation**	results in changes in protein sequences, and are under natural selection
**mutator phenotype**	mutations produced randomly throughout a population but don’t result in clonal populations
**heteroresistance**	denotes subpopulations with different resistance phenotypes

Terminology and associated definitions used herein.

Remarkably, the genome comparisons have unearthed a large correlation between the number of copies of a particular family of epithelial adhesins (EPA) and the pathogenicity of *C*. *glabrata*. This family of cell wall proteins mediates adhesion of *C*. *glabrata* cells to different substrates including host tissues and constitutes a well-known virulence factor in *C*. *glabrata* [7]. Interestingly, a closer phylogenetic analysis of the evolution of this family revealed that the larger size of the EPA repertoire in the pathogenic species corresponds to 2 independent expansions, one specific to *C*. *glabrata* and another one preceding the divergence of other members of the Nakoseomyces clade.

Genetic rewiring is not simply a feature of this yeast’s biology, but a major advantageous factor that contributes to its ability for resistance but also adhesion, another key trait of *C*. *glabrata*.

### 2: Cell wall as a dynamic organelle

The fungal cell wall is an essential organelle, providing physical strength, and limiting permeability, thereby retaining periplasmic proteins, and protecting cells from hostile host degrading enzymes and mechanical stress [8]. The cell wall also plays an important role in host–fungus interactions that facilitate the establishment of disease [8]. It is often the first point of contact between host and yeast cells and mediates interactions with the external environment through fungal adhesins and host receptors that, upon activation, trigger a complex intracellular signalling cascade [9].

Generally, the *Candida* species (spp.) cell wall is composed of glucans, chitin, chitosan, and glycosylated proteins (Fig 1) [8]. *C*. *glabrata* cell wall is a bilayered structure with a packed mannoprotein outer layer. Its organisation mirrors that of *S*. *cerevisiae*, but with a higher mannose/glucose ratio and 50% more protein The wall contains glycosylphosphatidylinositol (GPI) proteins as well as protein linked through a mild alkali-sensitive linkage to 1,3-B-glucan. The cells are surrounded by a 100- to 200-nm thick wall with a semitransparent inner layer and then surrounded by an electron-dense layer. Defined areas of abundant chitin occur at the septa, bud necks, and bud scars [7]. The relatively low levels of alkali-insoluble glucans imply fewer cross-links exist between glucan and chitin in *C*. *glabrata* composing approximately 20% of cellular dry weight, demonstrating a thinner glucan network and a dense outer layer of mannoproteins. Therefore, B-glucans in the cell wall may provide more effective masking from the host immune recognition by the receptor dectin-1 [7,10,11].

*C*. *glabrata* readily adheres to biotic and abiotic surfaces including epithelia and indwelling devises. A major contributor to this is the adhesive nature of *C*. *glabrata* governed by adhesion proteins positioned on cell surfaces. These proteins are grouped into families based on their N-terminal domains, with most literature concerning subfamilies I and II, containing the Epa, and Aed and Pwp proteins, respectively. The EPA family of adhesins is named for its role in host epithelial adherence; however, Epa1 and Epa7 function in endothelial cell adherence. This family is well studied in vitro, with reference strain genomes encoding up to 23 EPA genes. Additional adhesins include Aed1 and Pwp7, which mediate endothelial adherence and AWP adhesin-like wall proteins with varied functions roles in adherence to abiotic material. Reference strain genomes encode around 67 GPI anchored proteins, a high number compared to other fungi, and 44 of these are located in subtelomeric regions, in proximity to other adhesin-like sequences [12]. However, clinical isolates can harbour over a hundred adhesion-related proteins [13]. Adhesins are expressed during biofilm growth [14] where they contribute to adherence of the biofilm structure to a surface, and intracellular adherence. Biofilms characteristically consist of mixed populations: cells of different age, growth state, and nutrient requirements, as well as cells from distinct species, e.g., *C*. *glabrata* and *C*. *albicans* biofilms, and fungal and bacterial mixed biofilms. *C*. *glabrata* biofilms are difficult to treat and eradicate due to the intrinsic drug resistance and adhesiveness of the yeast combined with the dense nature of the biofilm structure. A further complication is presented by *C*. *glabrata* biofilms harbouring distinct ergosterol responses to azoles dependent on carbon source [15], indicating responses may be host niche dependent and further underlying the adaptability and flexibility of this pathogen. Thus, as a readily adhesive, intrinsically azole-resistant biofilm former, *C*. *glabrata* is particularly challenging to treat.

### 3: Rapid adaptation is key to survival

In their natural habitats, yeast cells undergo continuous metabolic shifts in response to changes in extracellular environmental challenges, and these can result in environment-dependent differential gene regulation. The rapid adaptation of gene expression through transcriptional regulation is a major mechanism of fungal response to rapidly changing environmental conditions. First described in *S*. *cerevisiae*, this is referred to as ESR (environmental stress response) [16]. ESR in *C*. *glabrata* is coordinated by Msn2, the main transcriptional response activator, in addition to Msn4 being crucial for resistance to various stresses, with the regulated transcriptional response to general stress including oxidative, nitrosative, osmotic, and heat. The activation of Msn2 and Msn4 causes their rapid accumulation in the nucleus, recruitment to chromatin, and activation of stress-responsive genes such as Hsp90. These gene products help the cell mount a response to the specific stress, allowing adaptation of metabolism, growth kinetics, and other processes in response to the stress. *C*. *glabrata* can quickly adapt to environmental changes as a commensal pathogen, with Msn2 and Msn4 working independently of each other, which is the converse of what is observed in *S*. *cerevisiae*, even under restrictive nutrient conditions, such as inadequate carbon, nitrogen, sulfur, or phosphorous levels [17]. *C*. *glabrata* will experience shifts in temperature, pH, serum, nutrients, and oxidation during establishment of disease within a host and persistence. *C*. *glabrata* grows optimally at 37°C and therefore is primed to thrive in the human host but can also grow under heat stress conditions up to 42°C. Temperature variability affects gene expression and can result in the induction or repression of genes linked to virulence. The wax moth larvae and invertebrate model of infection, *Galleria mellonella*, is susceptible to *C*. *glabrata* infection at 37°C but not at lower temperatures, implying genes required for virulence are switched on at 37°C [18].

Carbon metabolism is critical for the survival, propagation, and pathogenicity of many human fungal pathogens. Growth on carbon sources other than glucose, e.g., acetate, lactate, and ethanol inhibit both planktonic and biofilm growth in *C*. *glabrata*. Multiple studies [17,19] highlighted the necessity of glyoxylate cycle gene ICL1 for varied carbon source utilisation. During phagocytosis, microbes are housed in lysosomes—a specialised compartment where oxidative and nonoxidative mechanisms degrade and kill internalised microbes. *C*. *glabrata*, however, resists and persists within phagosomes, and metabolic flexibility may underlie this trait. Evidence suggests that growth in the presence of alternative carbon sources affects the phagocytosis of *Candida* species. Perhaps enhanced sustenance in starvation conditions allows intracellular replication within macrophages. *C*. *glabrata* cells are engulfed during bloodstream circulation, and *ICL1* promotes growth and prolonged survival of *C*. *glabrata* during macrophage engulfment. If *C*. *glabrata* can drive concealment within intracellular niches, it may also evade high concentrations of antifungals during treatment [20].

### 4: Antifungal drug resistance and the future of drug regimes

The antifungal repertoire is limited to 4 extant and 2 novel drug classes with azoles, echinocandins, and polyenes and flucytosine (a nucleoside analogue with proven utility in combination regimes for *Cryptococcus*) as the main classes. Azoles, such as fluconazole, inhibit sterol biosynthesis by targeting Erg11, a lanosterol demethylase and are considered first-line therapy. However, azole resistance is increasing, particularly in patients with prior azole exposure [21] through phenotypic adaptations mediating tolerance, mutations in Erg11, increased drug efflux via transcription, or the altered copy number of transport encoding genes. In cases of azole-resistant *C*. *glabrata*, echinocandins, such as caspofungin or micafungin, are often employed. Echinocandins function by inhibiting fungal cell wall biosynthesis and have demonstrated good efficacy against *C*. *glabrata* infections, even in cases of azole resistance [6]. However, the emergence of echinocandin resistance, via mutations in Fks1, is observed in clinical settings. Polyenes, such as amphotericin B, are reserved for severe *C*. *glabrata* infections or cases of multidrug resistance (MDR) [22]. Polyenes act by permeabilising fungal cell membranes, resulting in cell death (Fig 1); resistance to polyenes occurs via altered sterol biosynthesis and damage to plasma membrane integrity. However, significant and severe side effects are associated with current formulations of polyene, including nephrotoxicity and infusion-related reactions.

Management of *C*. *glabrata* infections is challenged by the emergence of MDR where the fungus is resistant to multiple classes of antifungals. In *C*. *glabrata*, MDR is a common occurrence and can be intrinsic or acquired. In such cases, combinational antifungal therapy may be required. Here, 2 or more antifungal agents with different mechanisms of action may reduce the likelihood of resistance emerging based on the development of resistance to multiple agents being less likely than development of resistance to single agent. Combination antimicrobial therapy holds promise as a treatment strategy for drug-resistant *C*. *glabrata* infections [23].

Understanding the mechanisms through which *C*. *glabrata* acquires resistance, including telomere dynamics, efflux pump upregulation, biofilm formation, and acquisition of resistance genes, is essential for developing effective strategies to combat MDR infections. Mutations in the pleiotropic transcription factor PDR1 result in gain-of-function to multidrug transporters such as CDR1, CDR2, and SNQ2 as well as adhesion and virulence genes [24]. The increased efflux results in decreased intracellular concentrations of antifungal agents (azoles) and consequently leads to resistance (Fig 1). Mutations in the ERG11 gene, which encodes the target enzyme for azoles (lanosterol 14α-demethylase), lead to reduced drug binding affinity and decreased susceptibility. The prevalence of azole resistance in *C*. *glabrata* varies geographically but can be as high as 15%, with prior azole exposure increasing susceptibility.

Resistance to echinocandins is largely conferred through mutations in hotspots of FKS genes, which encode glucan synthase [25]. These mutations result in changes to cell wall structure and thickness, reduced drug binding, and increased MIC (Fig 1). A third of echinocandin-resistant strains are also azole resistant, making polyenes a final therapeutic option available.

Compared to azoles and echinocandin, the polyene drug class rarely elicits drug resistance, potentially due to the fitness trade-off associated with polyene resistance [26]. The mode of action of polyenes is to permeabilise membranes, resulting in leakage from the cytoplasm (Fig 1) [19]. As previously discussed, biofilm growth encompasses high cell density, matrix production, altered growth rates, and nutrient limitation and permits protection of multiple cellular viability states deep within the biofilm; together, these features reduce drug penetration and facilitate resistance.

Several factors contribute to the development of MDR in *C*. *glabrata*. Firstly, the intrinsic characteristics of the fungus, such as its ability to rapidly acquire genetic changes and adapt to stress conditions, which promotes persistence within the host. Additionally, previous exposure to antifungal agents, prolonged therapy, and the formation of biofilm on indwelling medical devices create selective pressure, favouring the survival of resistant strains. Moreover, the frequent/improper use of broad-spectrum antifungals can contribute to the selection of resistant strains.

In addition to our deepening understanding of the biology of *C*. *glabrata*, improvement in diagnostic methods and speed will be crucial in identifying drug-resistant strains and informing treatment regimes. Surveillance of drug resistance patterns and resistance mechanisms will be critical in shaping clinical practice and policy. Finally, the development of novel antifungal drugs is underway. New drugs include Fosmanogepix, which inhibits GPI anchor biosynthesis through targeting Get1, and Ibrexafungerp, which inhibits cell wall biosynthesis. Novel therapeutics with new and varied mechanisms of action and/or combinatorial therapies that target multiple pathways simultaneously will be vital to circumventing resistance.

## Outlook

*C*. *glabrata* combines traits of genetic rewiring, cell wall modification, environmental adaptation, and intrinsic antifungal resistance to establish itself as a powerhouse of resistance against host and therapeutics. Going forward, if we are to succeed in overcoming the clinical problems presented by *C*. *glabrata* infection, it will be paramount to firstly understand the fungal mechanisms at play in resisting therapeutics and secondly create novel approaches that encompass multiple targets to tackling infections.
